# Cine magnetic resonance imaging in evaluating retroperitoneal leiomyosarcoma arising from the inferior vena cava

**DOI:** 10.1002/iju5.12660

**Published:** 2023-10-27

**Authors:** Taisuke Tobe, Tomoaki Terakawa, Yoshiko Ueno, Keitaro Sofue, Takuto Hara, Junya Furukawa, Jun Teishima, Yuzo Nakano, Kenichi Harada, Masato Fujisawa

**Affiliations:** ^1^ Department of Urology Kobe University Graduate School of Medicine Kobe Hyogo Japan; ^2^ Department of Radiology Kobe University Graduate School of Medicine Kobe Hyogo Japan; ^3^ Department of Urology University of Occupational and Environmental Health Fukuoka Japan

**Keywords:** cine MRI, inferior vena cava, leiomyosarcoma, retroperitoneal neoplasm, vascular neoplasm

## Abstract

**Introduction:**

Leiomyosarcoma of the inferior vena cava is associated with poor prognosis. Complete resection is the only curative treatment. We present a patient with this disease in whom cine magnetic resonance imaging was valuable in selecting the surgical strategy and mitigating invasiveness.

**Case presentation:**

A 68‐year‐old woman presented with right‐sided abdominal pain. Computed tomography revealed an 86 mm tumor in the right retroperitoneal space that extended into the inferior vena cava and reached superiorly to the right atrium. Percutaneous needle biopsy confirmed leiomyosarcoma. Cine magnetic resonance imaging demonstrated no adhesions between the tumor and the upper segment of inferior vena cava wall, nor with the right atrial wall, indicating resectability. Radical tumor resection was successfully performed without requiring thoracotomy.

**Conclusion:**

Cine magnetic resonance imaging appears to be useful in inferior vena cava leiomyosarcoma for evaluating adhesions between the tumor and vessel wall.

**Abbreviations & Acronyms**
3D: three‐dimensional
CT: computed tomography
FOV: field of view
GRE: gradient echo
IVC: inferior vena cava
MRI: magnetic resonance imaging
TE: echo time
TR: repetition time

**Keynote message**

Leiomyosarcoma arising from the inferior vena cava is rare and has a poor prognosis. Radical resection is the only curative treatment. Cine magnetic resonance imaging is valuable in evaluating adhesions between the tumor and vessel wall, which provides critical information to determine the appropriate treatment. Cine magnetic resonance imaging should be considered when planning the treatment of this disease.

## Introduction

Leiomyosarcoma arising from the IVC is rare and frequently located in the retroperitoneum. The reported 5‐year disease‐free survival is only 6%.^1^ Radical resection is the only curative treatment option, as radiation therapy and chemotherapy have limited efficacy.^1^, ^2^ When considering the optimal surgical approach for tumors involving the IVC, the assessment of tumor–IVC adhesion is crucial.

Cine MRI captures repeated images for a certain time, which provides detailed information regarding both anatomy and dynamic motion. The steady‐state free precession sequence enables the acquisition of high‐contrast images of blood without the use of a contrast agent. Cine MRI using steady‐state free precession is therefore valuable for assessing vascular and cardiac function.^3^, ^4^ Cine MRI is also commonly used to analyze joint motion^5^ and hemodynamics^6^ and in other applications.

We previously reported that cine MRI is useful to evaluate adhesion of IVC renal cell carcinoma thrombus to the IVC wall.^7^ Here, we present a patient with a leiomyosarcoma originating from the IVC in whom cine MRI was useful to evaluate adhesions between intraluminal tumor and the walls of the IVC and right atrium.

## Case presentation

**Fig. 1:**
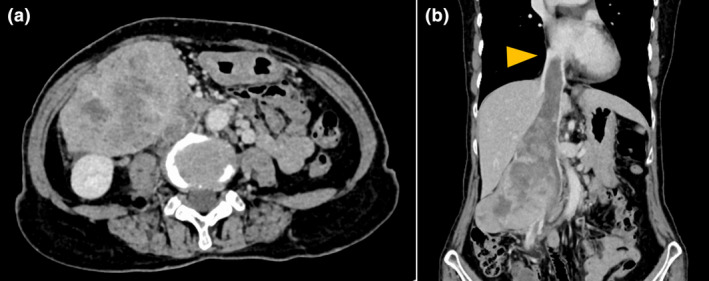
Contrast‐enhanced CT before surgery. (a) An axial view shows an 86 mm tumor in the right retroperitoneal space. (b) In a coronal view, the apex of the tumor thrombus (yellow arrowhead) extends into the right atrium.

**Fig. 2:**
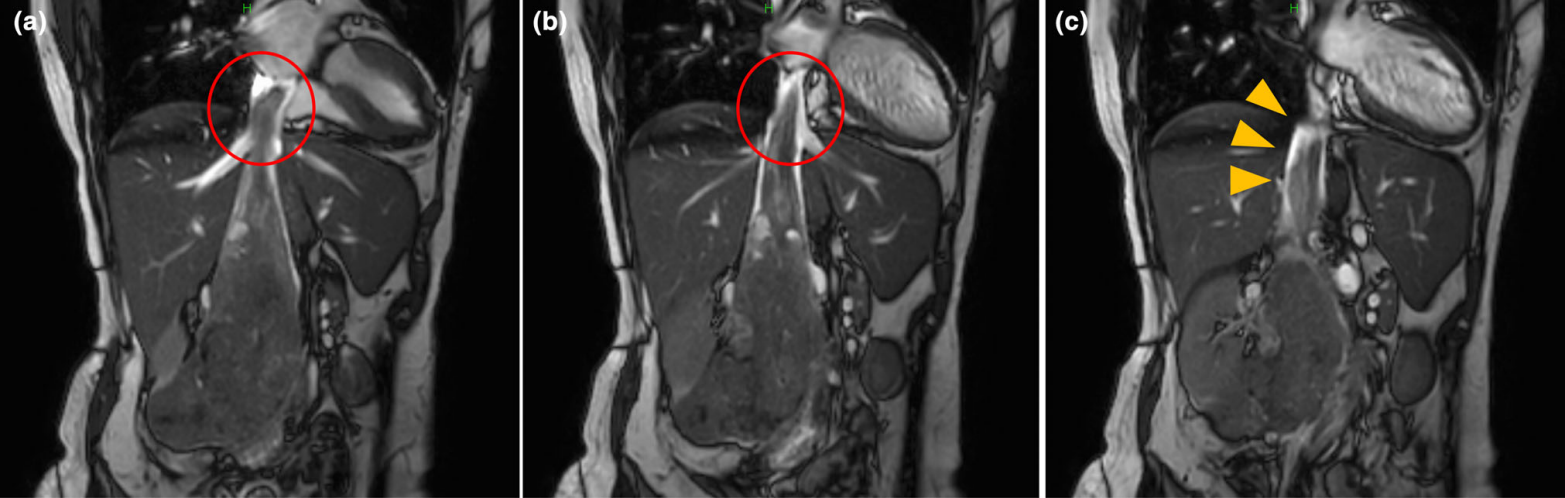
Coronal views of cine MRI before surgery showing the (a) tumor apex, (b) superior portion of the IVC, and (c) inferior portion of the IVC. The red circle indicates the apex of the tumor. Blood flow is seen between the IVC and the tumor (yellow arrows). The images confirm mobility of the top margin of the tumor within the right atrium and indicate the absence of adhesions between the tumor and the IVC wall.

A 68‐year‐old woman presented with right‐sided abdominal pain. Contrast‐enhanced CT revealed an 86 mm tumor in the right retroperitoneal space that extended into the IVC; superiorly, it reached the right atrium, and inferiorly, it extended to the confluence of the iliac veins (Fig. 1). On chest and abdominal CT, no findings suggestive of metastasis were observed. Percutaneous needle biopsy confirmed a diagnosis of leiomyosarcoma. Echocardiography revealed tumors in the IVC and atrium; however, adhesions could not be definitively assessed. We conducted cine MRI to assess tumor invasion into the IVC wall. The cine MRI comprised two different types of steady‐state free precession images, and the parameter details are presented in Table 1. Cine MRI demonstrated that the uppermost part of the tumor was located within the right atrium; this part of the tumor exhibited mobility and was not adherent to the right atrial wall. Furthermore, we observed blood flow between the tumor and the intraluminal wall of the upper segment of the IVC, and no adhesions were detected (Fig. 2). Based on these findings, tumor resection was considered feasible.

**Table 1:** MRI parameters

Variable	Parameter						
	Sequence	Acquisition plane	TR/TE (ms)	Matrix	Excitations (No.)	FOV (cm)	Section thickness (mm)
Deep breathing	3D‐GRE	Oblique coronal	3/1.3	192 × 204	1	36–38	8
Pulse synchronization	3D‐GRE	Oblique coronal	3/1.3	160–192 × 200–224	1	36	5

**Fig. 3:**
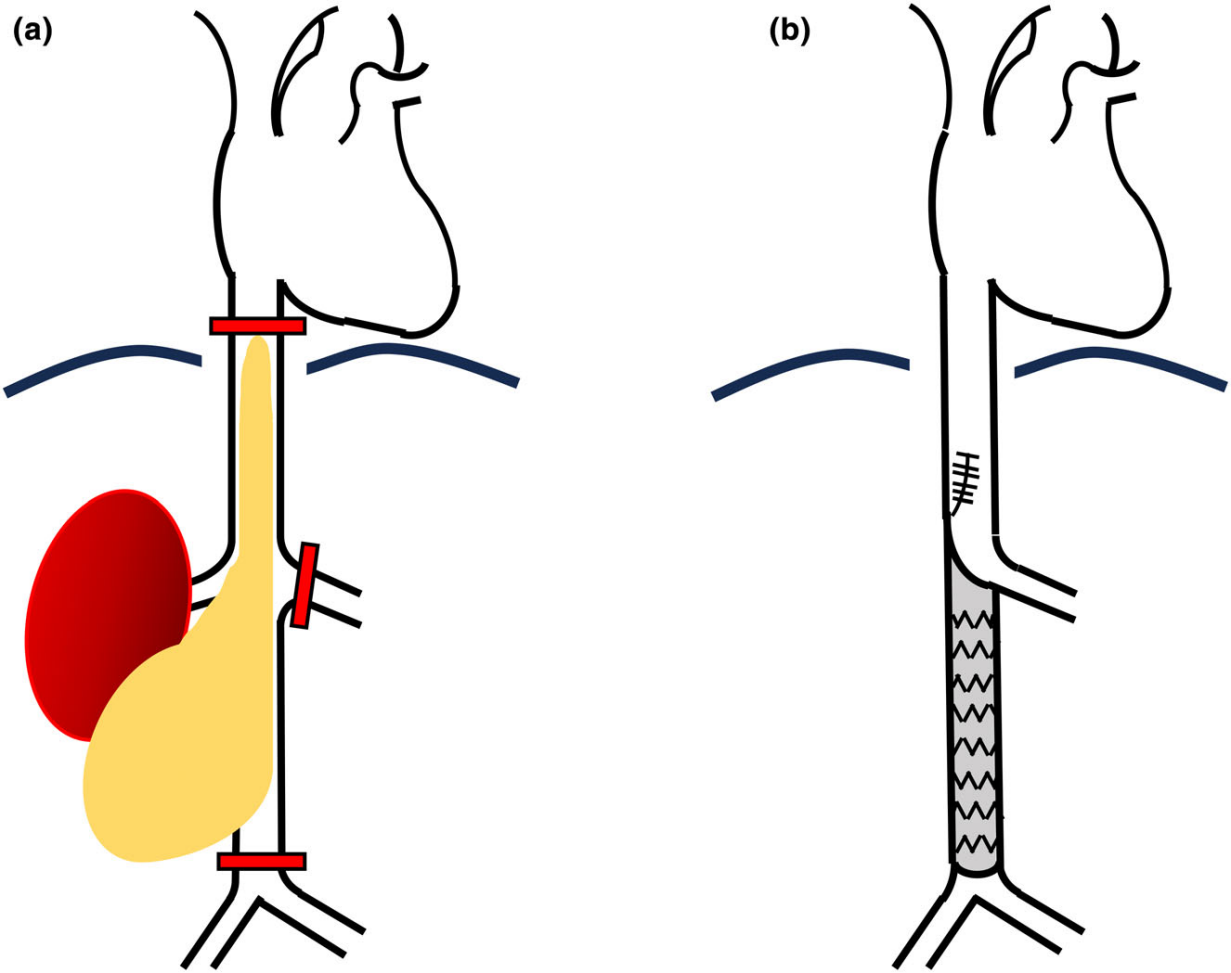
Schematic drawing of the intraoperative findings. (a) The three clamping sites, namely the superior aspect of the IVC near the right atrium, above the bifurcation of the common iliac veins, and at the left renal vein, are indicated with red bars. (b) The artificial vascular replacement was performed from the inferior border of the left renal vein to above the bifurcation of common iliac vein. A minor incision was made in the IVC superior to the right renal vein to withdraw the tumor. The incision was then closed with continuous sutures.

The surgery was performed via an abdominal approach. The tumor was contiguous with the IVC inferior to the renal vein. Following a diaphragmatic incision, palpation of the IVC near the right atrium revealed the mobility of the intravascular tumor. This affirmed our pre‐surgical predictions. The tumor's adherence to the right kidney necessitated en bloc resection. Clamping sites were obtained as shown in Figure 3, with the upper IVC clamp achieved by manually pressing down the tumor. After clamping, blood pressure remained stable at around 110/90 mmHg. The IVC inferior to the renal vein was strongly adherent to the tumor, so it was resected together, while the tumor superior to the renal vein was withdrawn. The artificial vascular replacement was performed from the inferior border of the left renal vein to the bifurcation. The operative time was 628 min, and the blood loss was 2750 mL.

The final histopathological diagnosis revealed pleomorphic leiomyosarcoma with a Ki‐67 index of 60% and negative margins. Some areas exhibited identifiable smooth muscle characteristics, while other regions were notably pleomorphic, suggesting highly undifferentiated zones. CT on postoperative Day 49 showed tumor recurrence in the right retroperitoneum. The subsequent CT on postoperative Day 65 showed metastases in the lung, liver, and right ischium. The patient died on postoperative Day 106.

## Discussion

In our patient, cine MRI was valuable for assessing the feasibility of surgical resection and showing that thoracotomy was not necessary. To the best of our knowledge, this is the first report demonstrating the benefit of cine MRI for evaluating intraluminal IVC leiomyosarcoma.

The primary treatment of IVC leiomyosarcoma is radical resection, which is the only approach with curative potential. Most cases become inoperable when the upper margin of the tumor extends beyond the hepatic vein.^8^ A case series of 22 IVC leiomyosarcomas reported that for tumors that extend beyond the hepatic vein, a thoracoabdominal approach is necessary; moreover, if the upper margin of the tumor extends into the heart, cardiopulmonary bypass is required.^9^ In our patient, surgical intervention without thoracotomy was deemed feasible based on the cine MRI findings, which proved to be true.

Cine MRI clearly demonstrated a lack of adhesions between the tumor and the vessel and right atrial walls in our patient. Various imaging modalities, including CT and MRI, can be utilized for diagnosing, staging, and monitoring IVC leiomyosarcoma.^10^ CT provides information regarding tumor size, spread within the IVC, and relationships with adjacent organs; therefore, it is the primary assessment tool. CT can also help discern the precise site of origin. Indistinct visualization of the IVC at the point of maximum contact with the tumor is an especially useful characteristic for clarifying an IVC origin, with a sensitivity of 75% and specificity of 100%.^11^ It has been reported that contrast‐enhanced MR venography provides crucial details regarding tumor location, growth pattern, morphology, extent, and collateral vessels and can distinguish tumor from non‐neoplastic IVC thrombus.^12^ However, as the information was obtained through contrast‐enhanced CT in our case, we did not perform imaging using this modality. Instead, we performed cine MRI to assess the venous wall invasion, which is a critical factor for surgical decision‐making. In our patient, cine MRI provided important information regarding tumor adhesion to the intraluminal wall. Previous reports have described using cine MRI to evaluate adhesions between lung cancer and the chest wall^13^ and between thoracic masses and cardiovascular structures.^14^ Cine MRI has also been used to investigate the movement of organs during radiation therapy for tumors of the liver, pancreas, esophagus, prostate, breast, and head‐and‐neck.^15^, ^16^, ^17^, ^18^, ^19^, ^20^ Cine MRI may have utility for evaluating tumor mobility and adhesions with adjacent organs. When considering treatment strategies for IVC leiomyosarcoma, cine MRI appears to be useful for evaluating adhesions between the tumor and vessel wall, which is important.

## Conclusion

We experienced a patient with IVC leiomyosarcoma in whom cine MRI was useful in determining the surgical approach. Cine MRI appears to be useful for evaluating adhesions between the tumor and vessel wall and should be considered as part of treatment planning to determine the appropriate approach.

## Author contributions

Taisuke Tobe: Conceptualization; visualization; writing – original draft. Tomoaki Terakawa: Conceptualization; writing – review and editing. Yoshiko Ueno: Writing – review and editing. Keitaro Sofue: Writing – review and editing. Takuto Hara: Writing – review and editing. Junya Furukawa: Writing – review and editing. Jun Teishima: Writing – review and editing. Yuzo Nakano: Writing – review and editing. Kenichi Harada: Writing – review and editing. Masato Fujisawa: Supervision.

## Conflict of interest

The authors declare no conflict of interest.

## Approval of the research protocol by an Institutional Reviewer Board

Not applicable.

## Informed consent

The patient provided consent for publication of this case report and any accompanying images.

## Registry and Registration No. of the study/trial

Not applicable.
